# Emborrhoid technique performed on a patient with portal hypertension and chronic hemorrhoidal bleeding as a salvage therapy

**DOI:** 10.1186/s42155-021-00278-8

**Published:** 2022-01-03

**Authors:** Filipa Alves e Sousa, Pedro Marinho Lopes, Inês Bolais Mónica, Ana Catarina Carvalho, Pedro Sousa

**Affiliations:** 1 Department of Radiology, Centro Hospitalar Universitário de Lisboa Central, Lisbon, Portugal; 2 Centro Hospitalar Universitário de Lisboa Central, Alameda Santo António dos Capuchos, 1169–050 Lisboa, Portugal; 3 Department of Radiology, Centro Hospitalar Vila Nova de Gaia/Espinho, Oporto, Portugal; 4 Department of General Surgery, Hospital Distrital da Figueira da Foz, Coimbra, Portugal; 5 Department of Gastroenterology, Centro Hospitalar Tondela Viseu, Viseu, Portugal

**Keywords:** Emborrhoid, embolization, hemorrhoidal disease, portal hypertension, rectal varices

## Abstract

**Background:**

Hemorrhoidal disease most commonly manifests itself with chronic rectal bleeding and, in its most severe and refractory forms, may lead to chronic anaemia with the need for recurrent blood transfusions. The main pathogenetic mechanism involved seems to be arterial hyperflux in the terminal branches that supply the hemorrhoidal plexus. It is based on this principle, that embolization of the superior rectal artery (emborrhoid technique) has recently re-emerged, with very promising results that support its feasibility, treatment efficacy, and safety.

**Case presentation:**

We report a case of a patient with both recurrent hemorrhoidal bleeding and portal hypertension with rectal varices, who underwent SRA embolization as a salvage therapy, with significant clinical improvement and no immediate or short-term complications.

**Conclusions:**

We believe that the positive results from our case raise the possibility that the emborrhoid technique could be effective and safe even in patients with portal hypertension, and that it would be clinically relevant to investigate this hypothesis on larger samples with a longer follow-up.

## Background

Hemorrhoids are normal anatomic vascular structures located at the submucosal layer of the anorectum, above (internal hemorrhoids) or below (external hemorrhoids) the dentate line. They are composed of a dense anastomotic arteriovenous network and connective tissue, known as corpus cavernosum recti, which contributes to the anal canal’s continence. While the pathogenesis of symptomatic internal hemorrhoids is not well understood, the main mechanism implicated is chronic increase in arterial flow with vascular congestion and hypertrophy of the hemorrhoidal cushions (Tradi et al. 2018; Lohsiriwat and Hemorrhoids 2012). Chronic rectal bleeding is the most common symptom, and may occur with or without associated hemorrhoidal prolapse(Lohsiriwat and Hemorrhoids 2012; Vidal et al. 2014). Embolization of the superior rectal artery (SRA), also known as the emborrhoid technique, has recently re-emerged as a potential alternative to surgery in patients with symptomatic hemorrhoidal disease refractory to conservative treatment(Vidal et al. 2014).

On a theoretically unrelated basis, patients with portal hypertension may experience rectal bleeding from submucosal rectal varices, which result from reopening of portal-systemic collateral veins in an attempt to diverge blood flow from the superior hemorrhoidal veins (portal venous system) to the middle and inferior hemorrhoidal veins (systemic venous system)(Lohsiriwat and Hemorrhoids 2012). The independent nature of hemorrhoidal disease and rectal varices is further supported by the fact that patients with portal hypertension and varices do not have an increased incidence of hemorrhoidal disease(Lohsiriwat and Hemorrhoids 2012). Massive variceal rectal bleeding is a rare occurrence but may result in life-threatening haemorrhage(Robertson et al. 2017). Treatment is usually directed at the underlying portal hypertension, with second-line/salvage therapies including rectal veins embolization, transjugular intrahepatic portosystemic shunt (TIPS) and surgery(Robertson et al. 2017).

We report a case of SRA embolization performed on a patient with both hemorrhoidal disease and portal hypertension with rectal varices, as a salvage therapy for recurrent rectal bleeding refractory to endoscopic injection sclerotherapy, endoscopic band ligation, inferior mesenteric vein embolization and TIPS.

## Case presentation

A 47-year-old male patient with alcoholic liver cirrhosis (Child-Pugh class B) and portal hypertension, entered the emergency department with acute rectal bleeding, hypotension (83/44 mmHg), and anaemia (haemoglobin levels of 5,6 g/dL). He initially received a blood transfusion and supportive care. Colonoscopy showed rectal varices (>20mm) and internal hemorrhoidal disease (grade III) with a low-rate active bleeding, which was treated with endoscopic sclerotherapy (polidocanol injection).

In the following 6 months, the patient experienced recurrent episodes of acute hemorrhoidal haemorrhage with chronic anaemia requiring blood transfusions (with haemoglobin levels ranging between 8,2 g/dL and 10,1 g/dL) that persisted despite several treatment procedures: a second endoscopic injection sclerotherapy, endoscopic band ligation, inferior mesenteric vein embolization, and TIPS.

Using the Hemorrhoidal Bleeding Score (Table 1) recently proposed by Fathallah et al. (2020) to objectively assess the patient’s clinical response, it could be inferred that none of the treatments performed had any significant clinical impact, since the resulting score persisted at 7 points throughout all of them (daily bleeding with anemia requiring blood transfusions and little discomfort).

Given the refractoriness of the hemorrhoidal disease and the patient’s comorbidities (which rendered him ineligible for surgical treatment), a multidisciplinary team decision was made to perform emborrhoid as a salvage therapy.

**Fig. 1:**
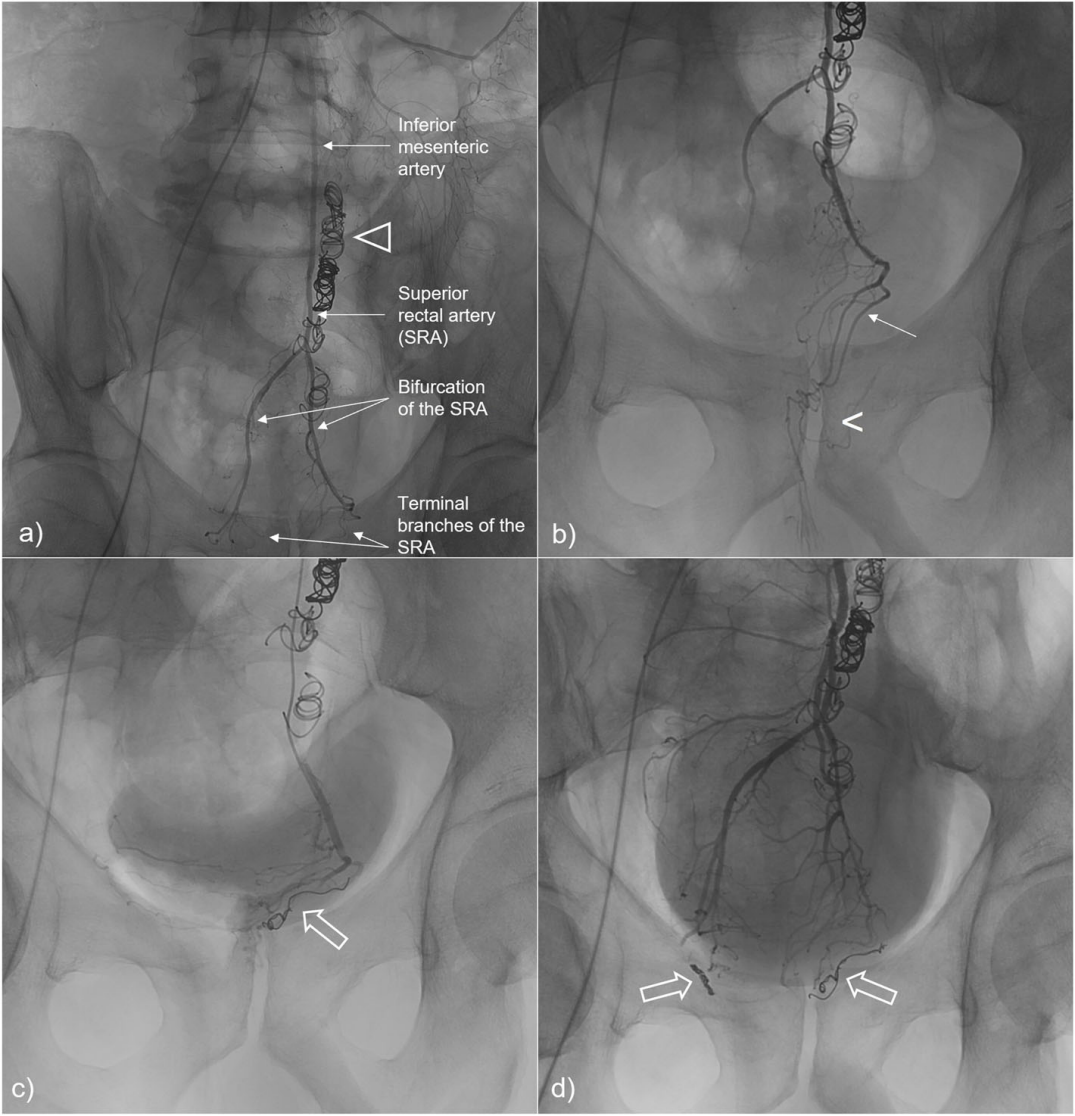
Endovascular coil-embolization of the superior rectal artery (SRA). Angiography of the inferior mesenteric artery **A**) displays normal anatomy, with opacification of the SRA and its terminal branches; also note the presence of coils in the inferior mesenteric vein (∆) from a previous venous embolization. Distal catheterization and opacification of the left branch of the SRA on **B**) allows better anatomic detail of the left terminal branches (arrow), selectively embolized with microcoils (open arrow) on **C**). Final control angiography after embolization of the left and right terminal branches of the SRA **D**) shows bilateral microcoils (open arrows) with upstream contrast stasis and absence of blood flow in the hemorrhoidal territory [projected at the level of the pubic symphysis (<) on image **B**)].

Embolization was performed under local anesthesia via a right femoral arterial approach. The inferior mesenteric artery was catheterized with a 5-F Simmons catheter. Digital subtraction angiography (DSA) was performed to identify all superior rectal artery branches supplying the corpus cavernosum recti. Each terminal branch was then catheterized using a 2.8-F microcatheter and embolized with 3mm-diameter microcoils. Technical success (defined as occlusion of all target branches from the SRA with interrupted blood flow in the hemorrhoidal territory) was achieved (Fig. 1).

The procedure was well tolerated, and there were no immediate or short-term complications.

Clinical improvement was assessed 6 weeks after the procedure using the Hemorrhoidal Bleeding Score (Table 1) which dropped from 7 to 2 (no bleeding, with anemia not requiring blood transfusions). The haemoglobin levels rose from 10,1 g/dL to 12,9 g/dL.

**Table 1:** Hemorrhoidal Bleeding Score

Variable	Score
Frequency	
Never	0
< 1/day or at each bowel movement	1
≥ 1/day or at each bowel movement	2
Type	
Never	0
Wiping +/- underwear	1
Toilet bowl	2
Anemia	
Never	0
Iron deficiency without anemia	1
Without transfusion	2
With transfusion	3
Discomfort	
Little or none	0
Moderate	1
Frank or permanent	2
Overall score	0 - 9

## Discussion

Results of current literature, from almost 250 patients analysed in a review article from Talaie et al. (2021), support the feasibility, efficacy, and safety of SRA embolization for hemorrhoids using microcoils, embolic particles or a combination of both. Results of immediate technical success rates (defined as the occlusion of all visible branches of the SRA above the pubic symphysis with closure of the corpus cavernosum recti plexus) range between 93 and 100%, and clinical success rates (classified as improved post procedural scores or well tolerated rectal bleeding) range between 63% and 94%, with no major complications. Recurrence of bleeding is the main reason for clinical failure, in most cases due to significant anastomosis with the branches from the middle rectal artery (found in up to 24% of patients), which can be treated by second procedures as needed.

Patient selection is the most heterogeneous variable among published series on emborrhoid technique, and this likely reflects the lack of well-established clinical indications and the uncertainty about which patients would benefit more from this procedure.

In a case report by Maiettini et al. (2018), embolization of the right terminal branch of the SRA was performed on a patient with rectal varices from portal hypertension and active hemorrhoidal bleeding on the right side, as a bridge treatment to TIPS placement. On retrograde phlebography of the superior hemorrhoidal veins after TIPS, there was a clear asymmetry on the rectal venous plexus congestion, substantially reduced on the (previously embolized) right side. These findings argue against a complete dissociation between hemorrhoidal disease and the increased downstream venous pressure on patients with rectal varices.

In a prospective study by Giurazza et al. (2020), five patients with chronic anaemia due to internal hemorrhoidal bleeding and cirrhotic portal hypertension were treated with the emborrhoid technique. The authors found it to be a safe and effective procedure, with clinical improvement in four out of the five patients, and no complications, at 3-month follow-up.

Hemorrhoidal disease and rectal varices may co-exist, and in most cases where portal hypertension is secondary to liver cirrhosis, there will be an increased risk of bleeding resultant from associated coagulopathy. Moreover, these patients frequently have multiple comorbidities and are not optimal candidates for conventional surgery.

Given the complex and not fully understood pathogenesis of hemorrhoidal bleeding in such patients with overlapping disease, and the safeness of the emborrhoid technique with no major complications reported in the literature, the authors argue that it would be pertinent to investigate the effectiveness of this procedure in patients with portal hypertension and refractory rectal bleeding.

## Conclusions

We reported a case of SRA embolization performed as a salvage therapy on a patient with both recurrent hemorrhoidal bleeding and portal hypertension with rectal varices, with technical success and clinical improvement. We believe that the positive results from our case and those from the above mentioned studies (Maiettini et al. 2018; Giurazza et al. 2020), should raise the possibility that the emborrhoid technique could be effective and safe even in patients with portal hypertension, and hopefully, this will encourage additional studies on larger samples with a longer follow-up.

## Data Availability

Not applicable.

## Abbreviations

DSA: Digital subtraction angiography
SRA: Superior rectal artery embolization
TIPS: Transjugular intrahepatic portosystemic shunt
